# Deep learning model for classifying shoulder pain rehabilitation exercises using IMU sensor

**DOI:** 10.1186/s12984-024-01343-8

**Published:** 2024-03-27

**Authors:** Kyuwon Lee, Jeong-Hyun Kim, Hyeon Hong, Yeji Jeong, Hokyoung Ryu, Hyundo Kim, Shi-Uk Lee

**Affiliations:** 1 Dept. of Rehabilitation Medicine, Seoul Metropolitan Government Boramae Medical Center, Seoul, South Korea; 2 Dept. of Physical Medicine & Rehabilitation, College of Medicine, Seoul National University, Seoul, South Korea; 3 Dept. of Graduate School of Technology and Innovation Management, Hanyang University, Seoul, South Korea; 4 Dept. of Intelligence Computing, Hanyang University, Seoul, South Korea

**Keywords:** Shoulder pain, Rehabilitation exercise, IMU sensors, Wearable sensors, Deep learning model, Deep neural networks (DNN), Machine learning, Exercise classification

## Abstract

**Background:**

Artificial intelligence is being used for rehabilitation, including monitoring exercise compliance through sensor technology. AI classification of shoulder exercise wearing an IMU sensor has only been reported in normal (i.e. painless) subjects. To prove the feasibility of monitoring exercise compliance, we aimed to classify 11 types of shoulder rehabilitation exercises using an AI (artificial intelligence) algorithm in patients with shoulder pain. We had the patients wear an IMU-based sensor, collected data during exercise, and determined the accuracy of exercise classification.

**Methods:**

Data were collected from 58 patients (27 males, 31 females, age range 37–82 years) diagnosed with shoulder diseases such as adhesive capsulitis and rotator cuff disease. 11 types of shoulder pain rehabilitation exercise programs were developed and repeated each exercise ten times per session while wearing an IMU sensor. The study applied the Rectified Linear Unit (ReLU) and the SoftMax as the activation function for hidden layers, the output layer.

**Results:**

The acquired data was used to train a DNN model using the multilayer perceptron algorithm. The trained model was used to classify 11 types of shoulder pain rehabilitation exercises. The training accuracy was 0.975 and the test accuracy was 0.925.

**Conclusion:**

The study demonstrates that IMU sensor data can effectively classify shoulder pain rehabilitation exercises, providing more appropriate feedback for patients. The model can be utilized to establish a system for remotely monitoring patients’ exercise performance. The use of deep learning in patient monitoring and rehabilitation has significant potential to bring innovative changes to healthcare service delivery.

**Supplementary Information:**

The online version contains supplementary material available at 10.1186/s12984-024-01343-8.

## Introduction

Shoulder joint disorders are estimated to account for 16% of the prevalence of musculoskeletal disorders and can cause pain, decreased muscle strength, numbness, paresthesia, and fatigue [1]. For most shoulder pain patients, conservative treatments such as nonsteroidal anti-inflammatory drugs (NSAIDs), joint injections, activity and work modification, physical therapy, manual therapy, botulinum toxin, and surgical intervention are generally considered first [2, 3]. After reducing pain through medication and injections, it is important to restore shoulder function through exercise therapy.

Exercise therapy is essential to normalize shoulder biomechanics and joint range of motion and can prevent disease recurrence [4–6]. To ensure exercise therapy is effective, compliance is crucial. Although medical professionals recommend that patients perform home-based exercises, patients often have difficulty performing home-based exercises consistently and independently, including the fact that home-based exercises may be limited without sufficient feedback or guidance. Previous research shows that up to 65% of patients do not adhere to home-based exercise programs, and approximately 10% fail to complete prescribed exercise therapy courses [7]. Without proper guidance and feedback, individuals may perform exercises incorrectly, risking injury or potential hazards. Recent advancements in technology have significantly impacted medical fields, offering new possibilities for objective monitoring and data recording in human movement [8–10].

Inertial Measurement Unit (IMU) sensors, combining accelerometers, gyroscopes, and magnetometers, have emerged as promising tools for capturing movement data during rehabilitation exercises. These sensors can accurately measure movements, directions, and positions in 3D space, providing detailed information on exercise performance [11–15]. With the use of IMU sensors, it is possible to monitor the movement of the patients during exercise.

Monitoring the movement during shoulder exercise therapy using an IMU sensor has been reported [16, 17]. However, the subjects in previous studies were healthy adults. To the knowledge of the authors, there are no previous studies in patients with disorders accompanied by shoulder pain. Patients, depending on their conditions and health status, require customized exercises, which limits the opportunity to utilize automatic exercise classification technologies.

The aim of this study is to train and test a Deep Neural Network (DNN) model to automatically classify 11 types of shoulder pain rehabilitation exercises using data collected from patients with shoulder pain. Using IMU sensor data, which captures three-axis accelerometer and gyroscope measurements of the shoulder joint during exercise, the DNN model is designed to identify different movement patterns associated with specific exercises.

## Methods

### Data acquisition

**Fig. 1:**
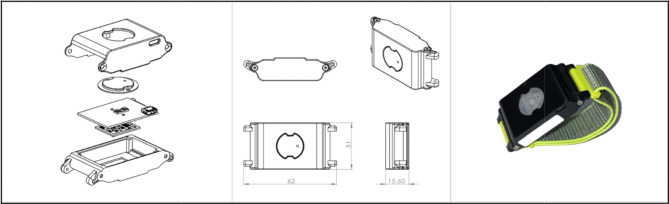
The schematics and actual image of the wearable sensor device. Engineered for wrist attachment, this device features an integrated IMU for real-time motion tracking and transmits data wirelessly to a computer

IMU sensor data was collected from participants in rehabilitation exercises for shoulder pain, The participants diagnosed with shoulder diseases such as adhesive capsulitis and rotator cuff disease were recruited from SMG-SNU Boramae Medical Center. The wearable sensor used for data collection (31 mm × 61 mm × 15 mm) was developed and used internally by the research team. It was designed to be worn like a wristwatch, can be fitted with a strap, and is equipped with an IMU sensor. An integrated sensor (MPU9250; InvenSense, San Jose, CA, USA) comprising a gyroscope (range, 2,000°/s), an accelerometer (range, 16 g), and a magnetometer (range, 49 G) was implemented as the inertial sensor for the module. The microcontroller was programmed to receive signals from the sensor, at a frequency of 100 Hz, through the SPI connection. The obtained angular velocity, acceleration, and magnetometer data were merged, and wireless Bluetooth module (PAN1321i; Panasonic, Osaka, Japan) was used to transmit the calculated data to a computer (Fig. 1).

**Fig. 2:**
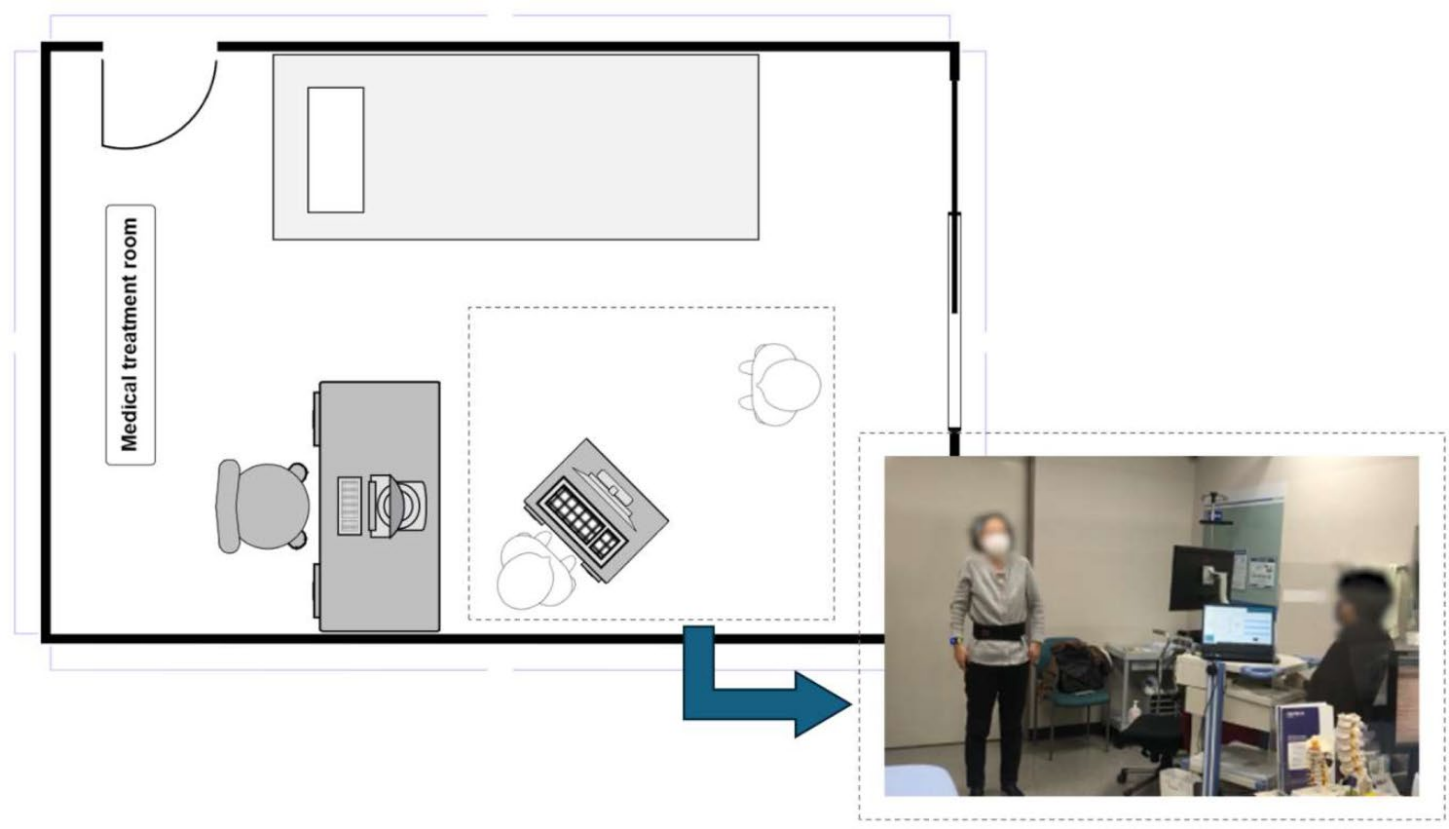
The experimental environment at SMG-SNU Boramae Medical Center. The data of the patients was collected in a medical treatment room

Through a visual program, the accelerometer and gyroscope of the IMU sensor can be monitored in real-time. The sensors were calibrated before data collection to ensure accurate measurements. The patients visited the hospital for rehabilitation exercise a minimum of 1 time and a maximum of 9 times, with 10 repetitions of the same exercise performed during each visit. To ensure accuracy and comprehension, 11 types of shoulder rehabilitation exercises were provided to patients via instructional videos (Table 1), and throughout the data collection process, the patients were monitored by clinicians or researchers to ensure proper exercise performance and reduce the risk of injury (Fig. 2).

**Table 1:** 11 types of shoulder rehabilitation exercise programs

No.	Shoulder pain rehabilitation exercise program
1	Posterior capsule stretching
2	Pectoralis minor stretching
3	Pendulum exercise
4	Lawnmower exercise
5	Shoulder abduction (with band)
6	shoulder extension (with band)
7	shoulder external rotation (with band)
8	shoulder internal rotation (with band)
9	shoulder external rotation (in side-lying)
10	Prone shoulder extension
11	Anterior deltoid strength

Rehabilitation exercise program performed by shoulder pain patients. Consists of exercise programs that can be performed by themselves in the hospital or at home

### Data preprocessing

The collected IMU sensor data was preprocessed to prepare it for training the deep neural network model. This preprocessing included several steps We identified the start and end times of exercise sessions to separate exercise data from rest periods, enabling our AI system to concentrate on pertinent and substantial data. Any erroneous or noisy data points were removed from the dataset by applying filtering program to identify and handle any inconsistencies in the sensor measurements.

### Feature extraction

Twenty-four distinct features were obtained by extracting four statistical indicators (minimum, maximum, average, and variance) from the x, y, and z axes of both acceleration and angular velocity.

### Data split

The dataset were divided into training, and testing sets. The training set was used to train the deep neural network model, the testing set was used to evaluate the final performance of the model.

### Deep neural network model (DNN)

**Fig. 3:**
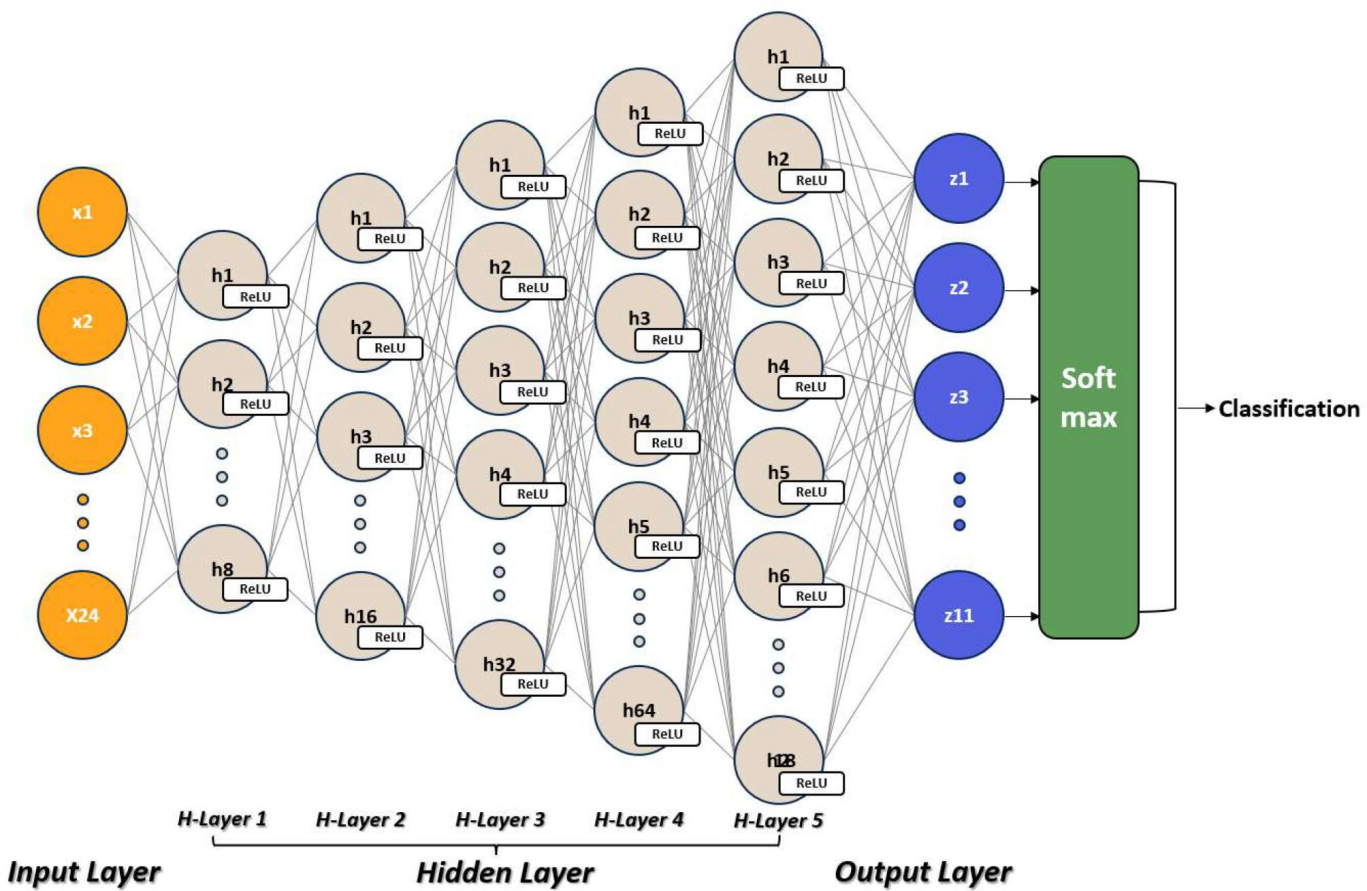
The Proposed DNN for shoulder rehabilitation exercise classification. The model consists of five hidden layers, with each layer having a varying number of nodes: the first with 8, the second with 16, the third with 32, the fourth with 64, and the last with 128 nodes. The Rectified Linear Unit (ReLU) function was used as the activation function for these hidden layers. For the output layer, a SoftMax activation function was implemented, allowing for effective multi-class classification

Deep Neural Network (DNN) is a potent tool that can closely determine elements impacting 3D positioning, as well as learn complex data patterns [18]. There has been recent interest in using multi-layer structured DNN to learn IMU sensor data, particularly in the fields of classification and numerical prediction [8, 9]. The DNN was constructed to classify the shoulder pain rehabilitation exercises based on the preprocessed IMU sensor data. The model included an input layer for the 24 features, and five hidden layers, each having 8, 16, 32, 64, 128 nodes, respectively, which aid the model in learning complex patterns and relationships in the data. The Rectified Linear Unit (ReLU) was used as the activation function for hidden layers, and the Softmax was used for the output layer [19, 20] (Fig. 3).

1. Input Layer: The input layer receives the preprocessed IMU sensor data, which acts as the model’s features.
2. Hidden Layers: five hidden layers are added to the model to capture the complex relationships and patterns in the data.
3. Output Layer: The output layer produces the classification predictions for the different shoulder pain rehabilitation exercises.
4. Activation Functions: The Rectified Linear Unit (ReLU) activation function is utilized in all hidden layers, facilitating efficient training by addressing the vanishing gradient problem and promoting faster convergence [19]. The Softmax activation function is employed in the output layer, converting the network’s output into a probability distribution over predicted output classes, and thus is suitable for multi-class classification tasks [20].

### Model training and evaluation

The preprocessed data was loaded and then split into training and test sets at an 8:2 ratio. Subsequently, a DNN model was trained by inputting the training data into the model. The preprocessed acceleration and angular velocity data from the test set were then input into the trained DNN model to predict the type of 11 shoulder rehabilitation exercises. The predicted workouts were compared to the patients’ actual data, and their accuracy was calculated using the accuracy score function from the scikit-learn library.

## Results

### General characteristics of participants

Fifty-eight participants diagnosed with shoulder disease participated in this study. Of the participants, 27 were male and 31 were female. The age range of the participants was 37 to 82 years, with the most prevalent age group being those aged 60 to under 70(53.4%). The average age of the participants was 60.5 years. To assess the severity of shoulder pain and disability, we used The Shoulder Pain and Disability Index(SPADI) questionnaire. It consists of 13 items that evaluate both pain and the impact of pain on various daily activities. The SPADI provides a total score ranging from 0 to 100, with higher scores indicating more severe pain and disability [21, 22](Table 2).

**Table 2:** General characteristics of shoulder pain patients (*N* = 58)

Category	N (%)	Mean ± SD (Min-Max)
Age(years)		60.5 ± 9.73 (37–82)
Less than 60y	25(43.1%)	
60y ∼ 70	22(53.4%)	
More than 70y	11(19.0%)	
Gender		
Male	27 (46.6%)	
Female	31 (53.4%)	
Height(cm)		164.3 ± 08.40 (152–185)
Weight(kg)		61.2 ± 10.36 (38–85)
SPADI score		43.9 ± 22.65 (5.4–80.8)

### Classification accuracy

When evaluating the performance of the developed deep learning model, two primary metrics were considered: train accuracy and test accuracy. The train accuracy reached 0.975, indicating the model’s proficiency in correctly classifying the shoulder rehabilitation exercises during the training phase. This high level of accuracy suggests that the model has effectively learned the patterns and distinguishing features from the training dataset, which consisted of 80% of the collected data. The model’s test accuracy of 0.925 suggests that it maintained high classification performance when exposed to the remaining 20% of the data, which it had not encountered during the training phase. The proximity of the test accuracy to the train accuracy indicates the model’s robustness and potential applicability in real-world scenarios, where maintaining accuracy on data beyond the training set is critical for the algorithm.

$$Accuracy=\frac{TP+TN}{TP+FN+FP+TN}$$

**Fig. 4:**
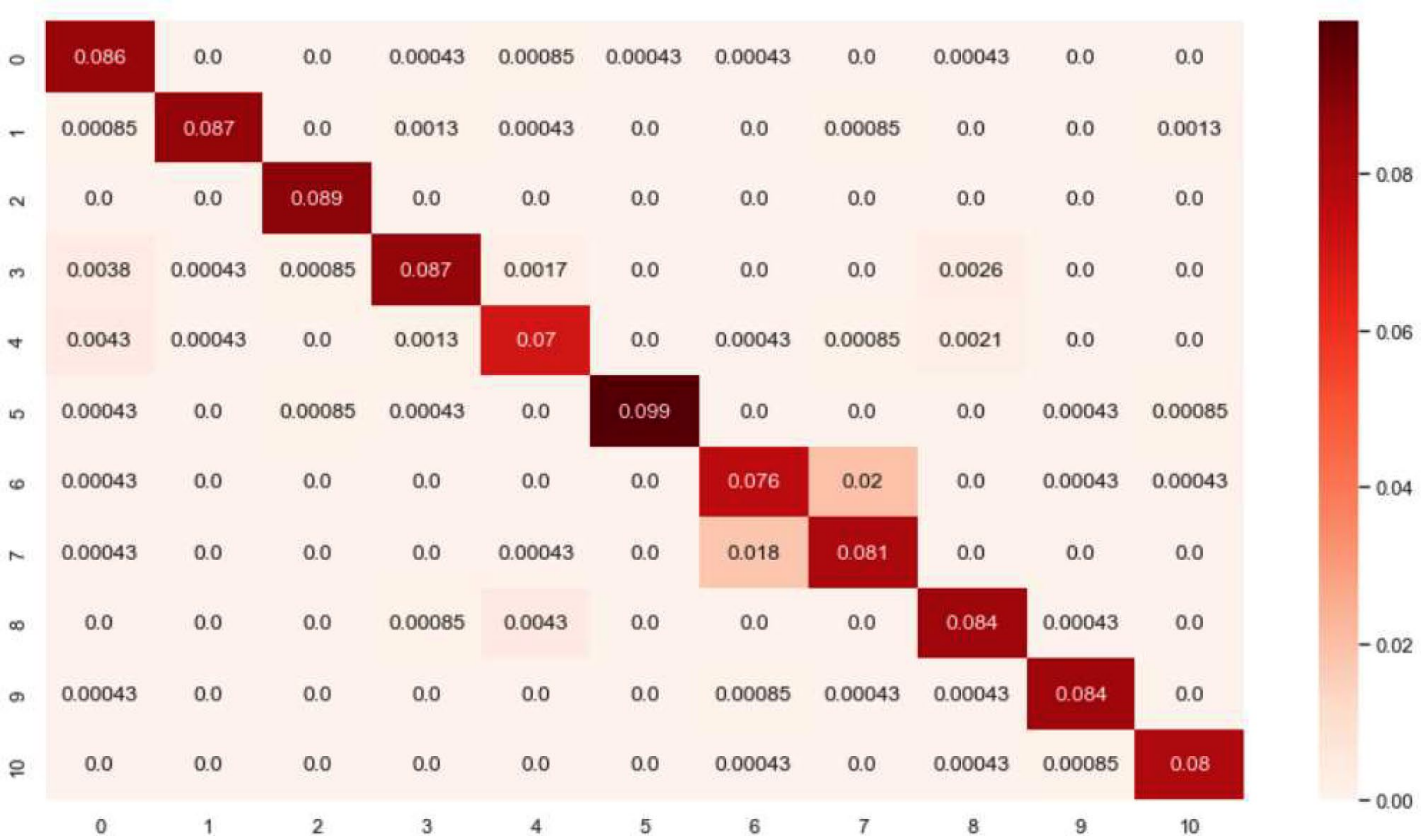
Heatmap confusion matrix showing multi-class classification results with all 11 shoulder rehabilitation exercises. Rows show the actual values(input), and columns show the predictions(output)

In this study, a heatmap was used for visualizing the model’s performance. The heatmap displays the level of agreement between actual values and predictive values, with the intensity of color indicating the degree of agreement. The diagonal direction of the heatmap is represented by dark colors, indicating that the model has made accurate predictions [10, 23]. This suggests that the model is highly accurate and reliable (Fig. 4). Out of the 11 exercises, the one with the highest prediction accuracy was ‘5) Shoulder abduction (with band)’ while the one with the lowest prediction accuracy was ‘4) Lawnmower exercise’. Notably, exercises ‘6) Shoulder extension (with band)’ and ‘7) Shoulder external rotation (with band)’ are also depicted, highlighting an interesting observation from the heatmap. The model exhibits cross-identification, predicting exercise 6 as 7 and vice versa. This misclassification between the two exercise types may suggest similar feature representations within the model’s learned parameters.

## Discussion

The research results presented in this paper show that 11 shoulder rehabilitation exercise programs performed with one wearable IMU sensor can be distinguished with 92.5% accuracy. The findings of this study are in line with previous research in Human Activity Recognition (HAR) that utilized IMU sensors to analyze human movements and distinguish them through deep learning models. Moreover, the significance of this research lies in its basis on actual patient data. The study focuses on patients with shoulder pain, rather than on routine exercises for healthy individuals. This provides valuable data that can be applied in future research on rehabilitation exercises for a broader range of diseases.

As the data were collected from patients with varying severity of shoulder pain, we expected that the pattern would be more complex and difficult to predict than the movements of the healthy general population. However, a data collection program was developed to mark exercises’ start and end points with specific numbers, making the data pre-processing much easier. As a result, it was possible to distinguish exercises with relatively high accuracy for patients.

**Fig. 5:**
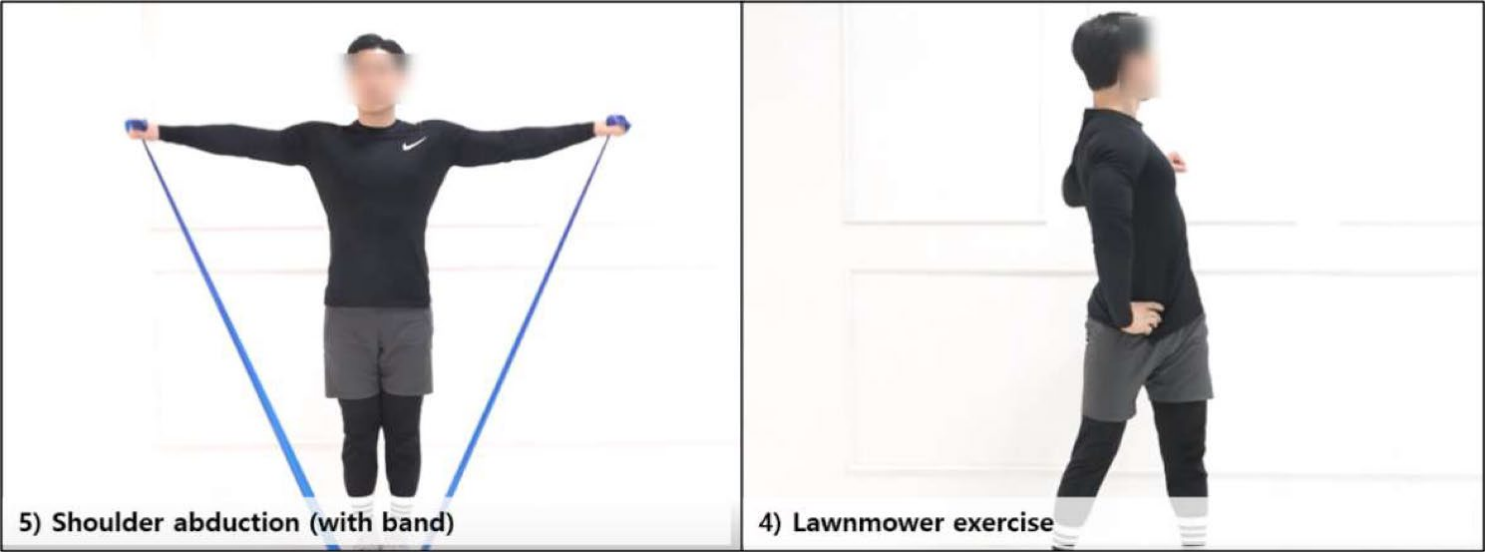
Image showing the exercise with the highest classification accuracy (no. 5), and the exercise with the lowest classification accuracy (no. 4) among 11 types of shoulder rehabilitation exercises

Furthermore, as observed in the heatmap results, exercise ‘5) shoulder abduction (with band)’ demonstrated the highest prediction rate compared to other exercises. This is presumably because the ‘5) shoulder abduction (with band)’ movement occurs within a single frontal plane and involves a wider range of motion, resulting in relatively uncomplicated IMU sensor data that is easier for the model to learn. Conversely, the exercise with the lowest prediction rate, ‘4) Lawnmower exercise’, involves movements across all three planes and allows for adjustment of motion range based on the intensity of the patient’s pain, which could have led to the collection of complex and diverse data (Fig. 5).

**Fig. 6:**
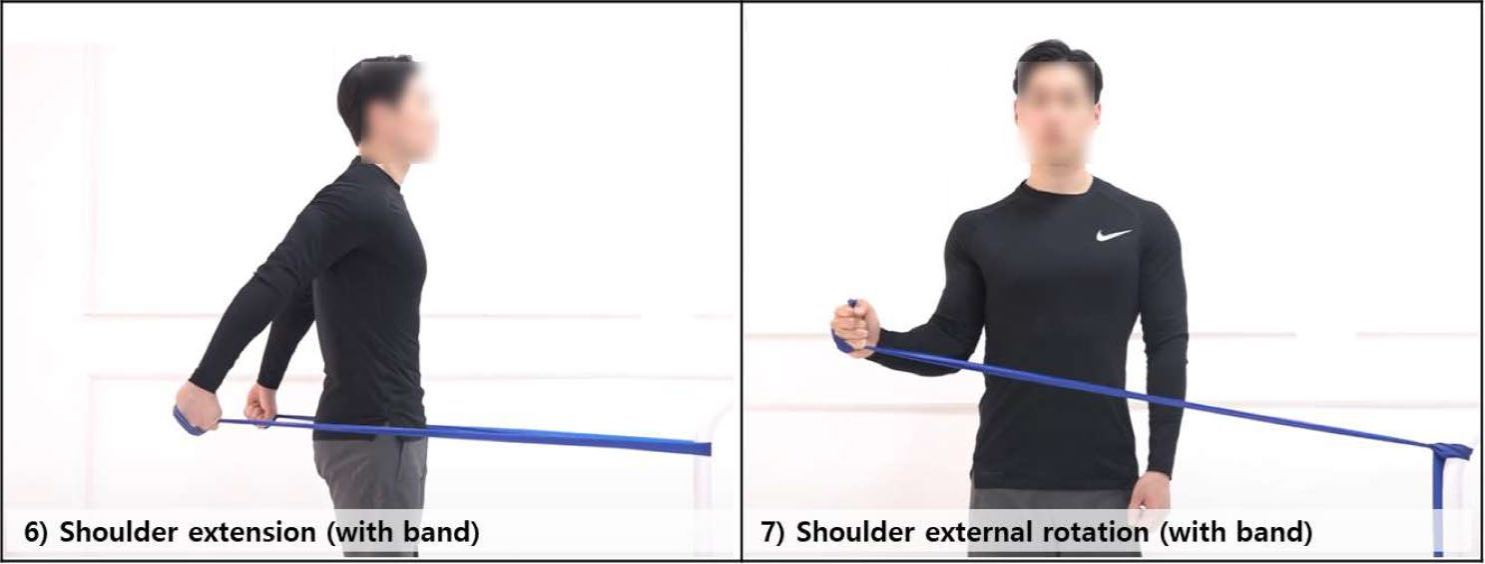
Images of exercise (no. 6) and (no. 7) as identified in the heatmap. The model displays instances of cross-identification, where it predicts exercise no.6 as exercise no.7 and vice versa

The Heatmap results of the classification experiment confirmed that exercise ‘6) Shoulder extension (with band)’ and ‘7) Shoulder external rotation (with band)’ were confused with each other with a probability of 0.018 to 0.02. This suggests that the data collected during the two exercises share similar features. Comparing the sensor data from the two exercises, we found that the sensor, when worn on the wrist, showed movements at remarkably similar positions, with comparable velocities, and along nearly identical trajectories (Fig. 6). An IMU sensor can track an object’s movement or estimate its posture by measuring its position, direction, and speed in three-dimensional space. Therefore, distinguishing between two different movements using only an IMU sensor can be difficult if the sensor’s position, direction, and speed values are very similar, even though they represent two different movements.

To identify the underlying factors contributing to these results, further research is necessary, taking into consideration variables such as exercise difficulty, range of motion, and duration of movements, to assess their impact on classification accuracy.

In hospitals, exercise prescriptions are given for rehabilitation treatment of musculoskeletal disorders, and patients can perform exercises under the direction of a physical or occupational therapist. However, once patients return home, it can be difficult for patients to continue exercising on their own and receive professional feedback when they return home. The study confirms that a single wristwatch-type sensor can automatically classify the type of exercise performed using a deep learning model. If this deep learning model is utilized to develop a home training service, combined with a mobile application or TV, patients will be able to exercise at home while wearing sensors. Medical professionals will have access to the patient’s exercise records. Based on this data, they can provide feedback and motivation to help patients continue exercising. The recorded information can also be used for future research on patients.

Consistent performance of rehabilitation therapy is extremely important. To prevent the chronicity of diseases and facilitate a return to daily life, devices that assist in performing accurate and regular movements are essential. Therefore, such technology is actively researched in the field of rehabilitation medicine and holds significant value for future investigation. The ability to monitor exercises could also be applied to telemedicine services like remote exercise prescriptions, which could result in positive social and economic effects.

However, this study has limitations. Because the DNN model was trained and validated using an 80:20 split within the same dataset, it necessitates validation against data collected in new environments. Additionally, while the amount of data used for training was not small, the fact that the subject group was composed only of patients with shoulder joint diseases experiencing shoulder pain limits the study by not utilizing data for various shoulder conditions. Since the treatment applied varies according to the specific diagnosis of shoulder disorders, there is a need for future research to categorize exercise programs more finely according to precise shoulder diagnoses.

## Conclusion

In conclusion, the application of deep neural networks to the classification of shoulder pain rehabilitation exercises using IMU sensor data shows promise for improving the monitoring and assessment of exercise performance. This technology has the potential to benefit both clinicians and individuals by optimizing exercise performance and improving outcomes in shoulder pain rehabilitation programs. Further research is necessary to enhance the model’s validity and applicability, firstly by expanding the dataset to include a wider range of shoulder disease severity and rehabilitation exercises for each severity. Additionally, investigating the model’s applicability in clinical settings, particularly in remote monitoring and patient adherence to prescribed exercises, is a vital next step. Our objective is to contribute to the development of an intelligent and personalized rehabilitation platform that can adapt to the unique needs of each patient. This will optimize recovery outcomes and enhance the quality of care.

### Electronic supplementary material

Below is the link to the electronic supplementary material.

Supplementary Material 1

Supplementary Material 2

Supplementary Material 3

Supplementary Material 4

Supplementary Material 5

Supplementary Material 6

## Data Availability

No datasets were generated or analysed during the current study.
