# Supplementary figures and images for: Deep learning model for classifying shoulder pain rehabilitation exercises using IMU sensor
Source: J Neuroeng Rehabil. 2024 Mar 27;21:42. doi: 10.1186/s12984-024-01343-8 (PMC10967210; doi:10.1186/s12984-024-01343-8)

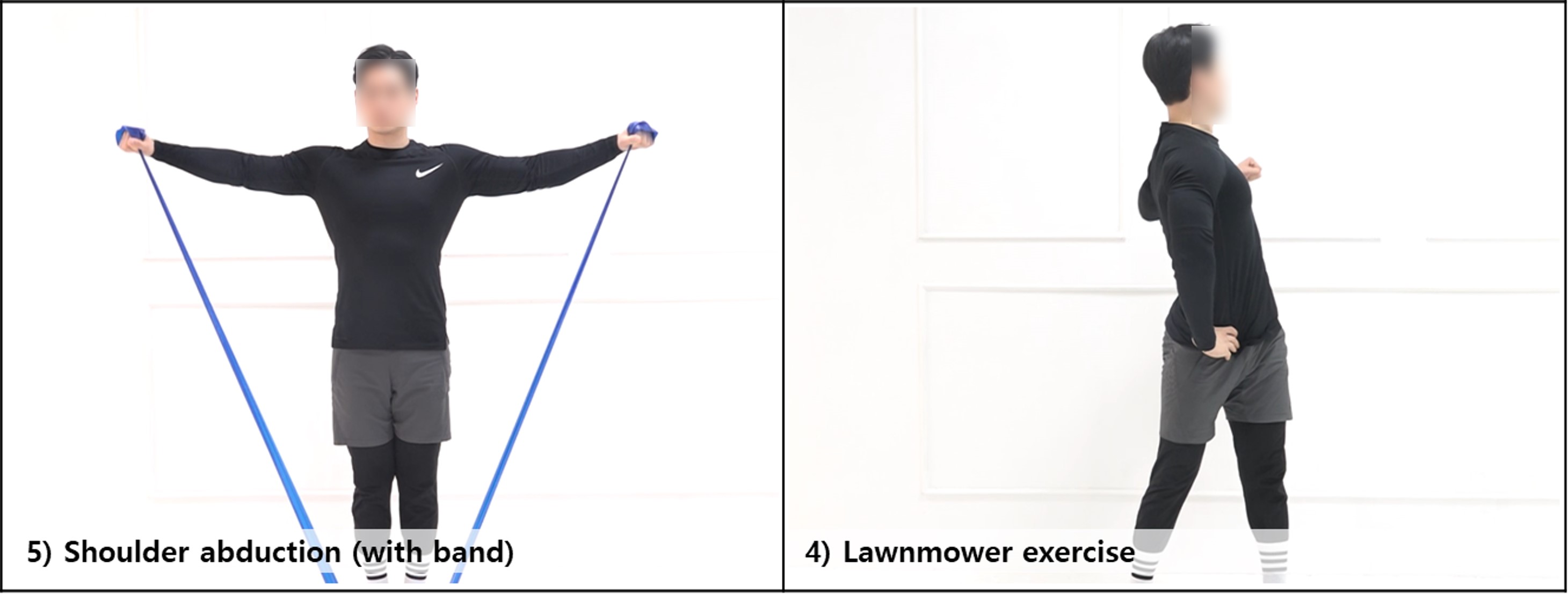

Supplement: Supplementary file 1 — Supplementary Material 1 [file 12984_2024_1343_MOESM1_ESM.jpg]

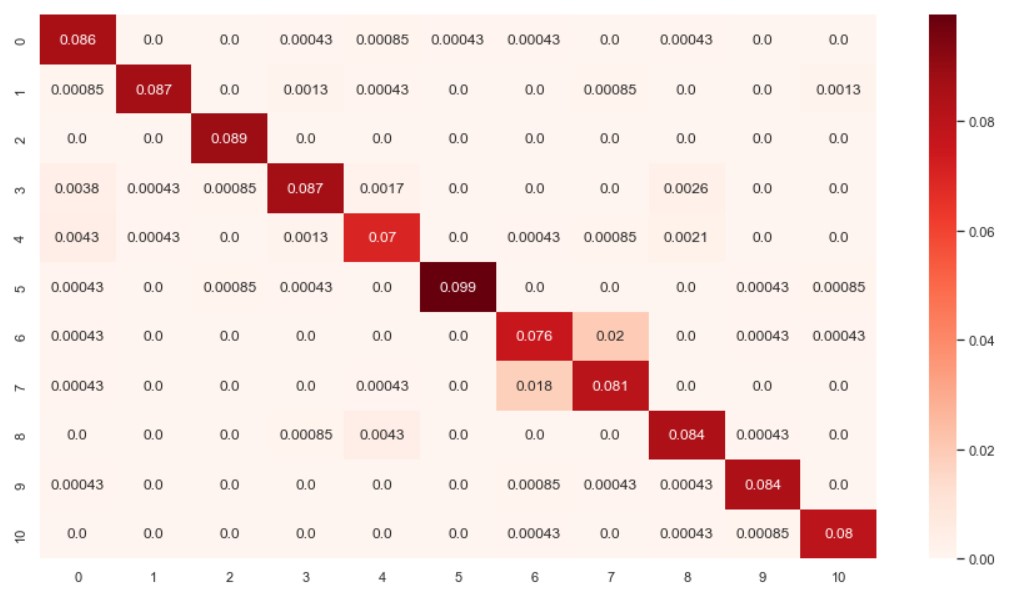

Supplement: Supplementary file 2 — Supplementary Material 2 [file 12984_2024_1343_MOESM2_ESM.jpg]

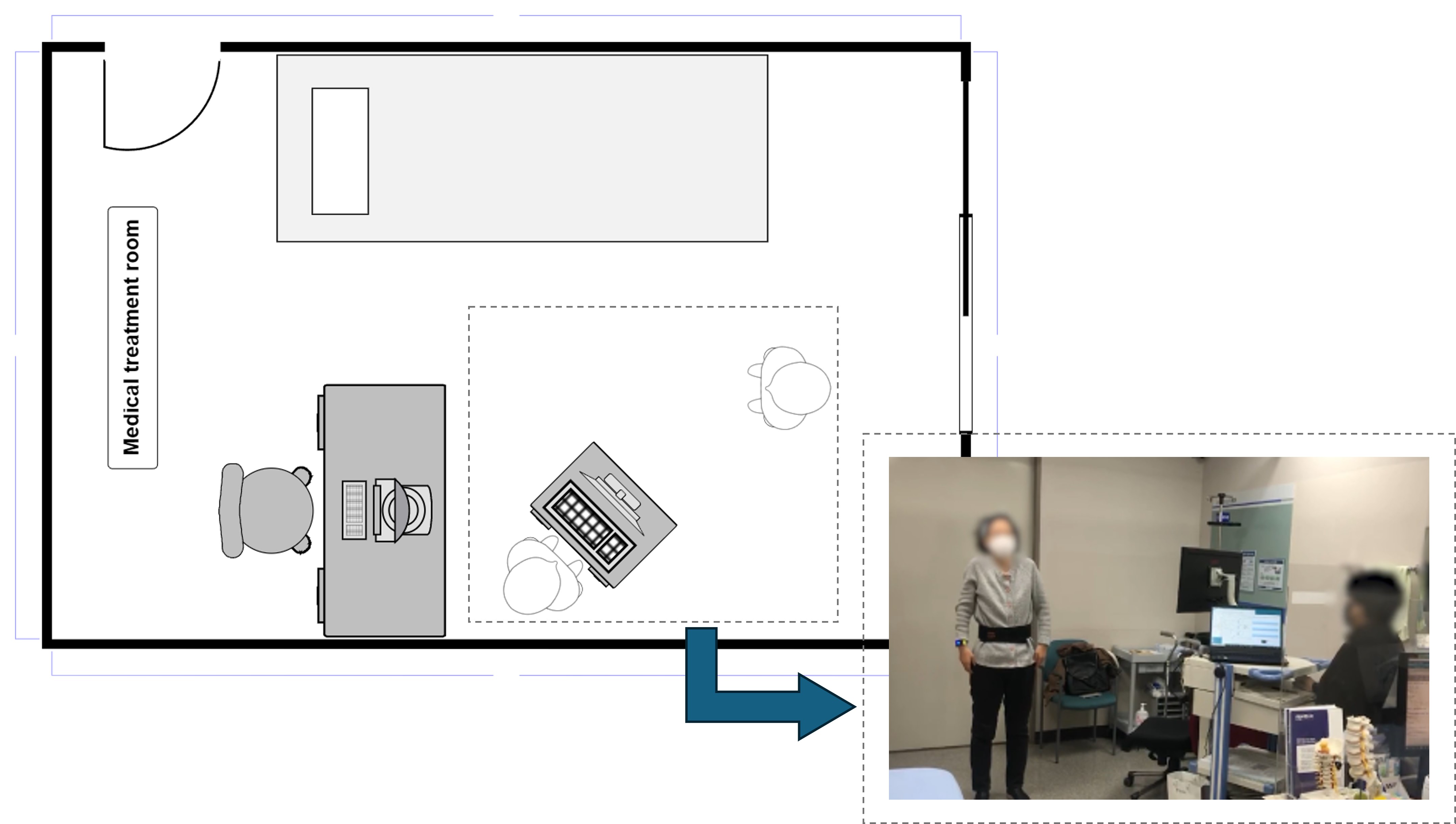

Supplement: Supplementary file 3 — Supplementary Material 3 [file 12984_2024_1343_MOESM3_ESM.jpg]

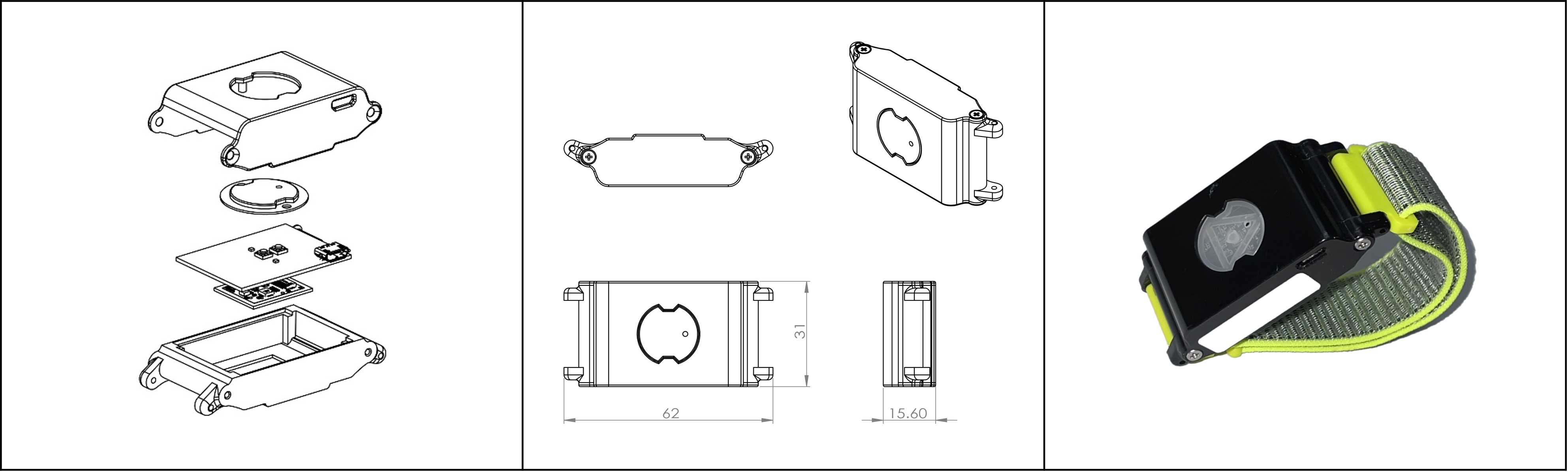

Supplement: Supplementary file 4 — Supplementary Material 4 [file 12984_2024_1343_MOESM4_ESM.jpg]

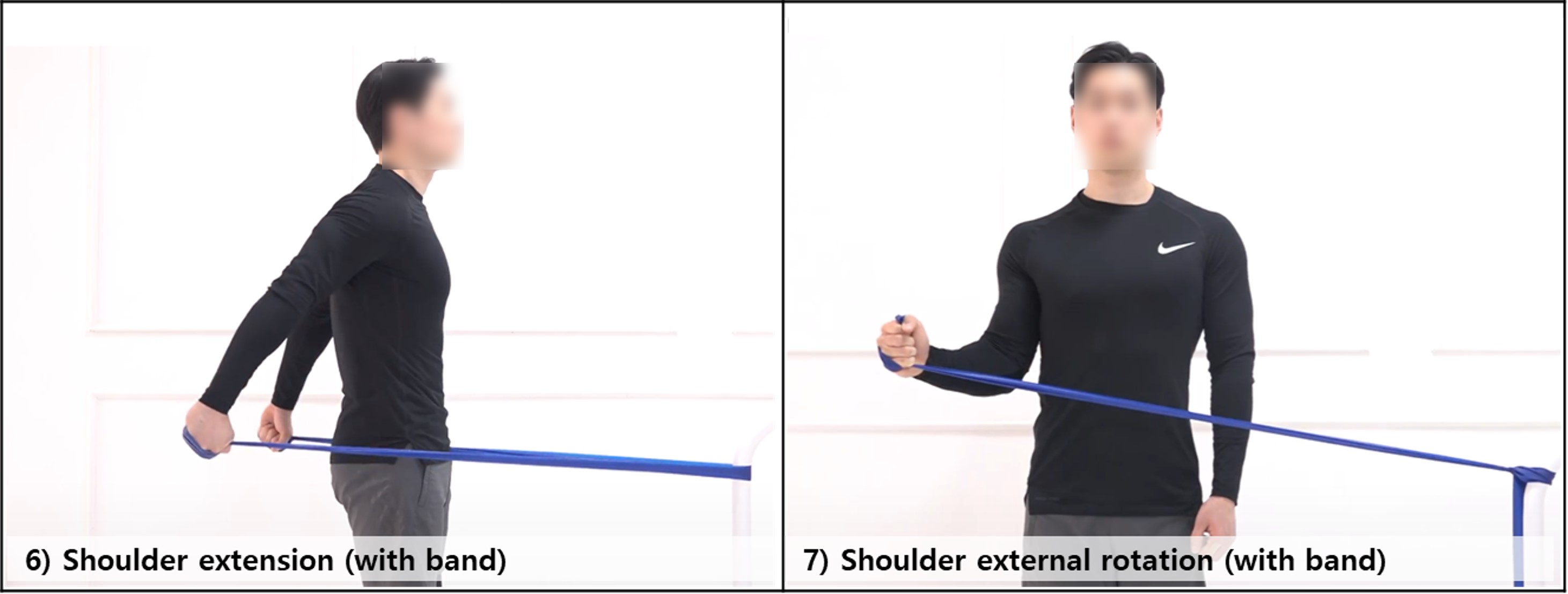

Supplement: Supplementary file 5 — Supplementary Material 5 [file 12984_2024_1343_MOESM5_ESM.jpg]

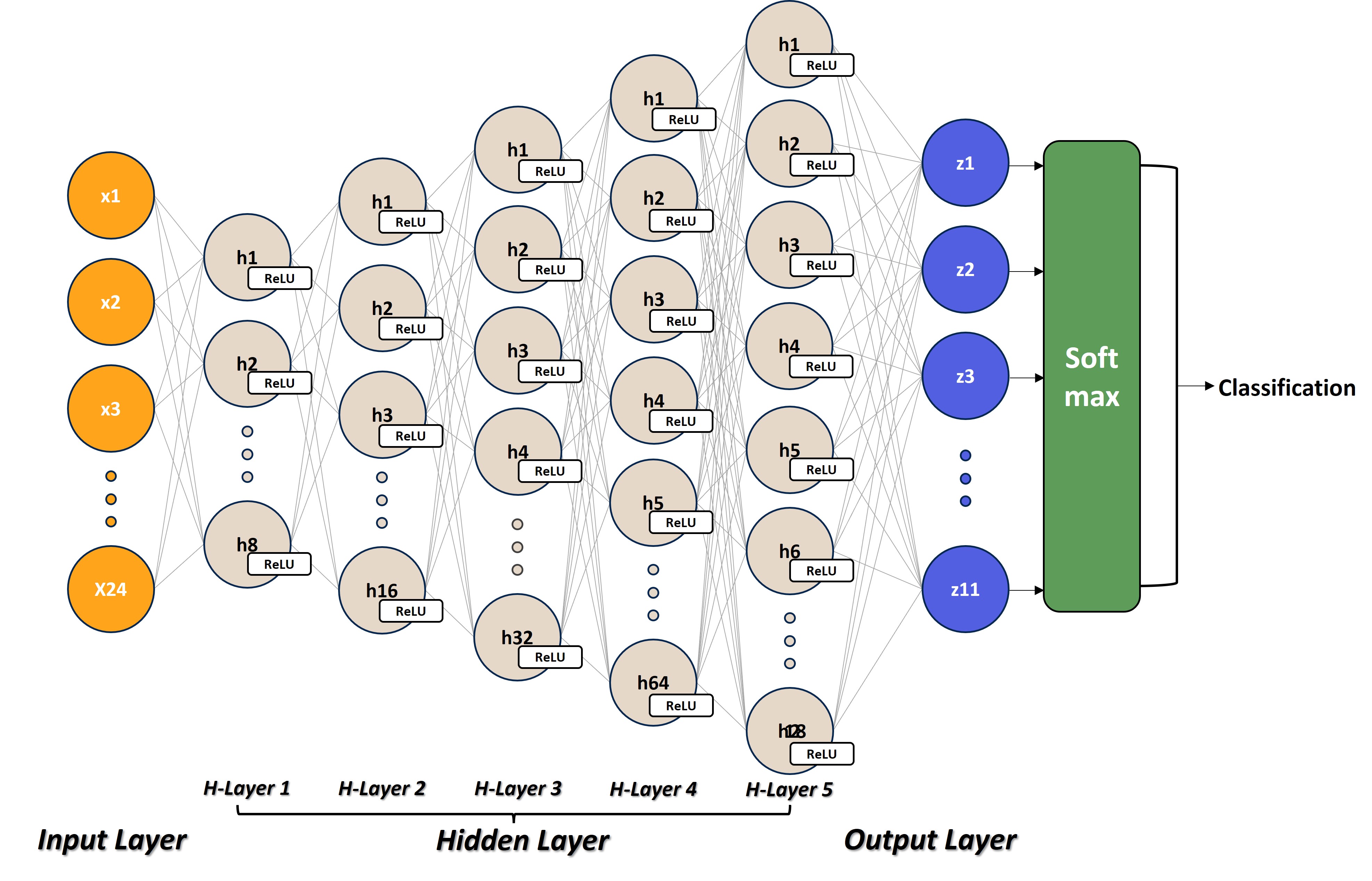

Supplement: Supplementary file 6 — Supplementary Material 6 [file 12984_2024_1343_MOESM6_ESM.jpg]
